# Ethical Use of Social Media and Sharing of Patient Information by Medical Students at a University Hospital in Saudi Arabia: Cross-Sectional Survey

**DOI:** 10.2196/57812

**Published:** 2025-03-24

**Authors:** Sara Farsi, Alaa Sabbahi, Deyala Sait, Raghad Kabli, Ghaliah Abduljabar

**Affiliations:** 1 Department of Anesthesia and Critical Care, Faculty of Medicine, King AbdulAziz University, KAUH, Jeddah, 21589, Saudi Arabia, 966 1264018335

**Keywords:** e-professionalism, professionalism, social media, medical education, curriculum development, privacy, confidentiality, ethics, patient confidentiality, cross-sectional, questionnaire

## Abstract

**Background:**

Social media (SM) has become an integral part of many medical students’ lives, blurring the lines between their personal and professional identities as many aspects of their medical careers appear online. Physicians must understand how to responsibly navigate these sites.

**Objective:**

This study aimed to identify how medical students use SM and their awareness and adherence to ethical guidelines of e-professionalism.

**Methods:**

This is a cross-sectional study delivered as an online voluntary survey to senior medical students at King AbdulAziz University Hospital in Jeddah, Saudi Arabia. We investigated how many students used SM, their privacy settings, their possible breaches of ethical standards, and their portrayal of their training institute online.

**Results:**

A total of 400/1546 (26%) senior medical students responded to our survey. Among the participants, 95/400 (24%) had public SM accounts, while 162/400 (41%) had both private and public accounts. As for breaches in e-professionalism, 11/400 (3%) participants posted a picture of a patient on SM without their permission, while 75/400 (20%) posted part of an excised organ or x-ray on SM without their permission, and 60/400 (16%) discussed a patient. With regards to sharing medical school information, 108/400 (29%) discussed an incident at their medical school, and 119/400 (31%) participants shared a lecture online without the presenter’s permission. Approximately 66% of the participants reported that they were unaware if their institution had a professional code of conduct for SM use, and 259/371 (70%) did not receive training on the professional use of SM.

**Conclusions:**

Medical students must be taught to recognize inappropriate online behavior, understand their role as representatives of their medical school, and know the potential repercussions of unprofessional conduct on SM. This could be accomplished by providing workshops, regular seminars on e-professionalism, and including principles of SM conduct in existing ethics courses.

## Introduction

Since its founding in 2006, the number of active users of Twitter (currently known as X) has increased to 237.8 million worldwide as of January 2023 [1]. Many medical students have grown up with online social media (SM) profiles. Studies conducted in Saudi Arabia have demonstrated that 75%‐87% of medical students use SM [2,3]. Owing to built-in camera-equipped smartphones, these students can now document their entire lives through pictures and videos and share them online like a public diary. Therefore, medical school is an integral part of their lives, and aspects of it are bound to find their way onto their SM profiles. However, do medical students understand the rules and implications of sharing that information online?

In the past decade, medical students have transitioned from discussing complex patient details with a few colleagues in the hospital’s breakroom to doing so with hundreds of “followers” worldwide. During the COVID-19 crisis, SM played a major role by building bridges across health care communities, allowing physicians and patients to connect worldwide, exchange experiences, access the latest health recommendations, and provide and receive emotional support [4-6]. SM has even been used as an educational resource, with studies showing that most students use it to access or share learning material; 30% do not even use a textbook [7-9]. In addition, students may also share patient encounters, conflicts between staff, recordings of lectures, and other occurrences on these SM sites. These medical student posts eventually become a reflection of their profession and institution. The images they share are not always complimentary. A cross-sectional study in the United States revealed 9 incidents of medical students posting negative information about their medical school online [10]. Furthermore, the same study revealed that 13% of those schools described a violation of patient confidentiality, and 4% of those incidents were reported by the patients or their families. Health care workers’ online posts have also led to dismissals and lawsuits [4,11,12]. Moreover, several articles document unprofessional behavior by medical students online, including drinking and illicit drug use [4,10,12,13].

We hypothesize that many medical school curricula emphasize disease management and patient care, which are undeniably important. However, they have not fully evolved to address the complexities of the modern social and digital landscape, leaving students underprepared to navigate these challenges effectively. This gap can inadvertently contribute to lapses in judgment because students face situations for which they may not have been adequately equipped. Against this background, our study aimed to determine whether medical students shared unprofessional content related to patients or their medical school that could impact public perception of their institution or profession. Additionally, we sought to assess their awareness of and adherence to ethical standards of e-professionalism. A further objective was to compare our findings within the context of Saudi culture to those reported in previously published Western studies.

## Methods

### Study Design

This is a cross-sectional study that includes senior medical students and interns at King AbdulAziz University (KAU). Medical school in KAU lasts 6 years in addition to an internship year. We defined senior medical students as those in their 4th to 6th years of training. This group was selected because the earlier years of medical education focus primarily on lecture-based and laboratory-based basic sciences, with no direct patient exposure. We developed a 2-part, 19-item survey and included 3 demographic questions (age, gender, and year of training). The question content and design were based on our primary and secondary research goals. We developed our research questions through an extensive review of the literature, aiming to identify common challenges, breaches, and issues faced by medical students and medical schools in the context of SM use [4,10,12,14]. We identified common issues among medical students, including the sharing of confidential patient information—both textual and visual—on SM, as well as the dissemination of negative encounters experienced in their hospitals. Additionally, this study found that numerous lecturers faced consequences for remarks or actions during lectures that were unknowingly recorded by students and shared publicly [15-17]. This prompted us to investigate the frequency of teaching sessions being recorded without the lecturer’s permission. Our survey questions were regarding sharing images of patients, parts of patients, colleagues, and lectures without permission. We also included questions on whether they discussed patients or incidents at their medical school online. To identify the effects students’ online behavior may have on their professional image, we included questions that addressed students’ profiles’ privacy or anonymity (eg, Do you use your real name? Is your profile picture a clear image of yourself?), and link to their profession (eg, Do you mention the name of your institution? Do you identify your profession?). We revised the survey to ensure that the final questions were relevant, contained appropriate wording, and appeared in a logical order. A questionnaire was developed using the website Survey Monkey. The results could only be accessed by the principal researcher under a password-protected online account. We shared the survey with 10 medical students from the target group to ensure that all respondents would similarly interpret the questions as well as the usability and technical functionality of the survey platform. After piloting the survey, some questions were modified (in the question “what social media platform do you use regularly?” we added options such as Telegram, Discord, and Reddit). We also changed the wording of some questions to improve clarity. These 10 students were not included in this study’s group. The final questionnaire consisted of 19 questions distributed over 4 pages (Multimedia Appendix 1). The questionnaire does not allow multiple responses for the whole duration of this study. If a student attempts to take the survey again using the same browser, they will see a message that they already took the survey. After final approval of the questionnaire and design, we invited senior medical students from years 4, 5, and 6 and the internship year to participate in the survey voluntarily through an open link. Members of the research team contacted the chief students of each academic year in person to explain the purpose and details of this study to share with all students in their year of training. Then, they sent the chief students a link to the survey via a WhatsApp (Meta Platforms, Inc) message to distribute to all students in their year individually. This message included the name and contact information of the principal researcher, the duration of the survey (3 min), and a link to the survey. The message also informed the students that their responses would be kept confidential, participation was completely voluntary, there was no incentive, and their evaluation and training would not be affected by their decision to participate in the study. We also included a QR code link on the last slide of anesthesia lectures given to the target group and invited the students to this study. The survey link was opened on August 10, 2022, and closed on June 16, 2023.

Descriptive statistics of variables were presented as counts and percentages to summarize the characteristics of the participants, including gender, age, and year of medical school. Chi-square tests assessed associations between categorical variables, and Fisher exact tests, as indicated. Univariate and multivariate logistic regression analyses were performed to identify predictors of cyberbullying exposure, with odds ratios (ORs) and 95% CI reported for each predictor. Variables included in the regression models were gender, age category, year of medical school, SM privacy status, time spent on SM, and training on the professional use of SM. Statistical analyses were performed using Stata (version 12.1 software, StataCorp LP). Cronbach α was used to measure internal consistency (0.75).

### Ethical Considerations

We obtained institutional review board approval to conduct the study from KAU’s Ethics Committee (reference #414-‐22). The online survey began with an informed consent statement that explained the purpose of the questionnaire and assured participants that all information would be kept confidential with no names or contact details recorded in the survey. Participation was entirely voluntary, with no reward for completing the survey and no penalty for choosing not to participate. The data were stored securely under password protection, and only the principal researcher had access.

## Results

We distributed the survey to 1546 participants, of whom 400 responded, yielding a response rate of 26%. Survey completion rate was 86% and both incomplete and complete surveys were used in analysis. Approximately half of the participants were sixth-year students (194/400, 49%), and two-thirds were women (246/400, 62%), as illustrated in Table 1. Snapchat was the most used platform (287/400, 72%), followed by Twitter (275/400, 69%) and Instagram (256/400, 64%). Facebook was the least used platform (8/400, 2%), and only 8/400 (2%) of the participants reported not using any SM platform at least once a week.

**Table 1.** Characteristics of the participants (N=400).

Characteristic	Value
Gender, n (%)	
Male	154 (38.5)
Female	246 (61.5)
Age (years), n (%)	
18‐20	3 (0.8)
21‐25	378 (94.5)
26‐30	18 (4.5)
>30	1 (0.2)
Year of medical school^a^, n (%)	
Fourth	16 (4)
Fifth	142 (35.5)
Sixth	194 (48.5)
Intern	48 (12)
Platform used at least once a week, n (%)	
Facebook	8 (2)
TikTok	184 (46)
Snapchat	287 (71.8)
Twitter	275 (68.8)
Instagram	256 (64)
Reddit	32 (8)
Discord	30 (8)
Telegram	247 (61.8)
Own YouTube channel	25 (6.3)
None	8 (2)

a Has missing value for 1 participant.

Only 95/400 (24%) of the participants had public SM accounts, whereas 162/400 (41%) had a combination of private and public accounts. Most of the participants (307/400, 77%) used their real names on SM, and one-third used their own photos for their profile image (118/400, 30%). Approximately half of the participants used SM for more than 3 hours a day (180/400, 47%), whereas only 15/400 (4%) used it for less than 1 hour a day (Table 2). Most of the participants used SM for entertainment (340/400, 85%); some used it for networking with other professionals worldwide (91/400, 29%) and for staying in touch with family and friends (300/400, 75%).

**Table 2.** Description of social media use among the participants (N=400).

Variable	Participants
Privacy status of social media account, n (%)	
Public	95 (23.8)
Private	139 (34.8)
Some public, some private	162 (40.5)
Do not use social media	4 (1)
Privacy practices in social media use, n (%)	
Use of real name on social media	307 (76.8)
Use of a clear photo of self as a profile image	118 (29.5)
Identify as a King AbdulAziz University student	76 (19)
Identify as a medical student	127 (31.8)
None of the above	69 (17.3)
Time spent on social media, n (%)	
Less than 1 h/d	15 (3.9)
1 h/d	27 (7.1)
2 h/d	71 (18.6)
3 h/d	88 (23.1)
More than 3 h/d	180 (47.2)
Reason for social media use, n (%)	
Networking with other medical students or professionals worldwide	91 (22.8)
Keeping in touch with family or friends	300 (75)
Providing medical advice and advocacy	30 (7.5)
Entertainment	340 (85)
Medical education	172 (43)

Institution-related SM use practices are presented in Table 3. Regarding the professional use of SM, only 125/371 (34%) of the participants said they were aware that their institution had a professional code of conduct for SM use. Additionally, just 112/371 (30%) recalled having received training in the professional use of SM. Approximately one-third of the participants reported checking SM while rounding on patients (138/382, 36%), discussing an incident that occurred at their institution online (108/371, 29%), or uploading the content of a lecture or workshop online without the lecturer’s permission (119/382, 31%). Only 11/380 (3%) posted pictures of patients on SM after obtaining the patient’s permission, while 75/381 (20%) posted pictures of parts of a patient (x-ray, excised organ, etc) on SM without obtaining their permission.

**Table 3.** Number of participants who answered yes to questions regarding institution-related social media use practices, code of conduct, and training on social media use among the participants.

Survey question	Number of respondents^a^, n	“Yes” response, n (%)
Does your institution have a professional code of conduct or protocol that addresses the use of social media?	371	125 (33.7)
Did you receive any training during medical school or residency on the rules and regulations for the professional use of social media?	371	112 (30.1)
Checked your social media account while rounding on patients	382	138 (36.1)
Posted a picture of a patient on social media without their permission	380	11 (2.9)
Posted an image of part of a patient (including excised tumors or organs) or a radiographic image of a patient without a patient’s permission	381	75 (19.7)
Posted an image of a work colleague or senior without their permission	382	25 (6.5)
Uploaded a video or image of a lecture or workshop online without the lecturer’s permission	382	119 (31.2)
Discussed an incident that happened in your institution online	371	108 (29)
Discussed a patient you saw at your institution online	372	60 (16.1)

a Some of the values do not add up to the total because of missing values.

Furthermore, many participants used apps to search for medical information (Table 4). The most common apps were YouTube (314/340, 92%; Google LLC) and AMBOSS (301/340, 75%; AMBOSS GmbH), followed by Osmosis (250/340, 74%; Elsevier) and UpToDate (235/340, 70%). Wikipedia (35/340, 10%; Wikimedia Foundation, Inc) and Medline (40/340, 12%; Medline Industries, LP) were the least commonly used sources.

**Table 4.** Applications used among the participants to look up medical information (N=340).

Application	Participants, n (%)
YouTube	314 (92.4)
Medline	40 (11.8)
UpToDate	235 (69.1)
Wikipedia	35 (10.3)
AMBOSS	301 (75.3)
Osmosis	250 (73.5)
Other:	40 (11.8)

Other sources were BMJ, Board and Beyond (McGraw Hill), Dr. Najeeb (DrNajeebLectures.com), MedED (PW MedEd), Kaplan, Google, ChatGPT (OpenAI), Lecturio, OnlineMedEd, Mayo Clinic (Mayo Foundation for Medical Education and Research [MFMER]), Medscape (WebMD LLC), Medicosis Perfectionalis, Radiopaedia, Healthline (Healthline Media LLC), NCBI StatPearls (National Library of Medicine), Orthobullet (Lineage Medical, Inc), WikEM, Telegram (Telegram FZ-LLC), and Twitter (X Corp).

The associations between SM use practices and gender are presented in Table 5. Women were more likely than men to have private SM accounts (96/248, 39% and 43/154, 28%, respectively; *P*<.001) and were less likely than men to use a clear photo of themselves for a profile image (45/248, 18% and 73/154, 47%, respectively; *P*<.001).

**Table 5.** The association between cyberbullying, social media privacy status, social media privacy practices, and gender among the respondents (N=400).

Survey question	Male	Female	*P* value
Experienced cyberbullying, n (%)			.13^a^
No	118 (80.3)	194 (86.2)	
Yes	29 (19.7)	31 (13.8)	
Social media privacy status, n (%)			.001^b^
Public	53 (34.4)	42 (17.1)	
Private	43 (27.9)	96 (39)	
Some public, some private	56 (36.4)	106 (43.1)	
Do not use social media	2 (1.3)	2 (0.8)	
Privacy practices in social media use, n (%)			
Use of real name in social media	117 (76)	190 (77.2)	.77^a^
Use of a clear photo of self as a profile image	73 (47.4)	45 (18.3)	<.001^a^
Identify as a King AbdulAziz University student	30 (19.5)	46 (18.7)	.85^a^
Identify as a medical student	46 (29.9)	81 (32.9)	.52^a^
None of the above	29 (18.8)	40 (16.3)	.51^a^

a Chi-square test.

b Fisher exact test.

Of all the participants, 60/400 (16%) reported experiencing cyberbullying. In univariate analyses, participants with private SM accounts were less likely to experience cyberbullying compared to those with public accounts (OR 0.40, 95% CI 0.2-0.9). Additionally, those spending more than 3 hours per day on SM had significantly higher odds (OR 3.36, 95% CI 1.0-11.5) of experiencing cyberbullying compared to those spending 1 hour or less per day. Same findings were found in multivariate analyses but became borderline significant (all had confidence intervals that narrowly include the null value).

Table 6 presents the association between patient privacy practices among the participants and the privacy status of the SM accounts. Participants who reported posting an image of part of a patient (including excised tumors or organs) or a radiograph were more likely to have a mix of public and private accounts (39/75, 52%) than public (21/75, 28%) or private accounts (15/75, 20%; *P*<.001). Among the participants who reported posting an image of a colleague without obtaining permission, 12/25 (48%) had a public account, whereas 8/25 (32%) and 5/25 (20%) had mixed and private accounts, respectively (*P*<.001). Moreover, participants who uploaded the content of a lecture online without the lecturer’s permission were more likely to have a public account (37/119, 31%) than mixed (54/119, 45%) or private (28/119, 24%) accounts.

**Table 6.** Association between the privacy status of social media accounts and patient privacy practices.

Survey question	Privacy status of participants who answered “yes” to social media accounts			*P* value
	Public, n (%)	Private, n (%)	Mixed, n (%)	
Posted a picture of a patient on social media without their permission	5 (45.5)	2 (18.2)	4 (36.4)	.24
Posted an image of part of a patient (including excised tumors or organs) or a radiographic image of a patient without a patient’s permission	21 (28)	15 (20)	39 (52)	.01
Posted an image of a work colleague or senior without their permission	12 (48)	5 (20)	8 (32)	.01
Uploaded a video or image of a lecture or workshop online without the lecturer’s permission	37 (31.1)	28 (23.5)	54 (45.4)	.004
Discussed an incident that happened in your institution online	29 (26.9)	36 (33.3)	43 (39.8)	.68
Discussed a patient you saw at your institution online	20 (33.3)	20 (33.3)	20 (33.3)	.14

## Discussion

Our study reveals that a substantial portion of students frequently share medical school-related content online, with notable instances of ethical breaches such as discussing patients and posting images without consent. While most published studies examine unprofessional online content posted by students, we investigate how often aspects of their medical school that may affect public perception appear on their profiles. These results underscore the urgent need for enhanced e-professionalism training. Of the students who responded to our survey, 246/371 (66%) were unaware of institutional guidelines addressing the use of SM, and 259/371 (70%) felt they had not received training on the professional use of SM. However, most students in our study (389/400, 97%) refrained from posting images of a patient online despite not having received e-professional training. Probably, they recognized this as a breach of the well-known Hippocratic oath.

This study did uncover some breaches of professionalism. Of the students who participated in our survey, 60/372 (16%) discussed patients online, and 75/381 (20%) posted pictures of a patient’s excised organ or radiological image. Their intention was likely to share clinical experiences and demystify rare medical conditions, possibly unaware that they may be violating privacy regulations. Even if the information is deidentified using the Health Insurance Portability and Accountability Act’s “safe harbor” technique, it may not be anonymous [18]. If the clinical scenario is unique enough, the patient might be recognized or even appear in the local news [19]. Furthermore, patients or their families may find the case description or the public’s online comments hurtful or offensive. In response to several incidents, the Saudi Ministry of Health developed guidelines requiring physicians to obtain the patient’s consent before sharing their images or health information online or submitting it to a journal [20,21]. Any breach of these guidelines carries a hefty penalty.

When a personal profile is linked to a profession or institution, it becomes part of its public image, brand, and professional identity. In our study, in the participants’ SM profiles, 127/400 (31.8%) indicated that they were medical students, and 76/400 (19%) indicated the name of their university. Among them, 91/400 (22.8%) used their accounts to network with other professionals worldwide, making them representatives of their institutions and professions. Furthermore, students used YouTube (314/340, 92%) as a clinical reference more than websites with verified peer-reviewed content, such as UpToDate (235/340, 69%) and AMBOSS (301/340, 75%). Among our participants, 162/400 (41%) had both a public and private profile (one profile may have reflected a professional identity and the other a private one). Female students in our study are more likely than male students to have private profiles (96/248, 39% and 43/154, 28%, respectively; *P*<.001) and less likely to use a clear photo of themselves for their profile image (45/248, 18% and 73/154, 47%). This gender difference could stem from the conservative culture in Saudi Arabia or the universally higher vulnerability of women to online criticism and cyberbullying [22,23]. Regardless of privacy settings, medical students must be cautious when deciding what to post on their SM profiles since the content can be leaked.

Among the students, 119 (37 with a public profile) uploaded recordings of lectures or workshops without obtaining the presenter’s permission. This behavior is concerning, as comments and expressions made by educators or attendees may be taken out of context by worldwide viewers. Educators often tailor teaching material to their intended audience. They also ensure the cultural appropriateness of their expressions and comments while observing the audience’s social norms. If educators are aware that their work will be shared with a wider online audience, they may decide to change their appearance, behavior, and lecture content. They may also choose to avoid comments that may cause controversy among other groups. These fears have led many UK universities to implement lecture capture policies to manage the recording and dissemination of lecture content [24]. The policies address concerns related to intellectual property rights, emphasizing the need for the lecturer’s consent before recording and sharing materials. Furthermore, in our study, 108 students (29 with a public profile) discussed online incidents that had occurred at their institutions. These incidents may have been unintentionally misrepresented by these students. Studies have proven that eyewitness accounts are not always accurate [25]. Additionally, these incidents may have been exaggerated online for comedic or dramatic purposes or unintentionally reveal confidential patient information. Unfortunately, public criticism of these online posts will be directed at the students’ profession and medical school [26,27].

Our findings contribute to the growing body of literature on medical students’ SM use by highlighting specific behaviors and awareness levels in the context of the Kingdom of Saudi Arabia. While many of our results align with previous studies, notable differences were also observed. For instance, similar to a French study, most of our students used YouTube for medical education [28]. Although, our numbers (314/340, 92%) are much higher than those in France (504/762, 66%). However, only 172/400 (43%) of our students use SM for education compared to 42/63 (67%) of Canadian students in 2015 [29]. Additionally, 60%‐92% of medical schools in the United States have also experienced unprofessional online behavior by medical students [10,14]. Most students in both regions reported using restrictive privacy settings, with only 20%‐37% of US students failing to do so [30,31]. However, unlike our American counterparts, our students are less likely to use a clear profile photo, with female students being less likely than male students to do so. By contrast, an American study found that 57% of medical students had a clear profile photo, with females being more likely to display one than males [31]. While in India, 80% of students used a clear profile photo [32]. This discrepancy may reflect cultural differences in SM use. In Saudi Arabia, where our study was conducted, cultural norms and societal expectations may influence their online behaviors. These findings emphasize the importance of contextualizing SM behaviors within cultural and geographical frameworks to develop targeted interventions that address both universal and region-specific challenges.

This study’s findings are consistent with the results of other studies suggesting that medical school curricula should be regularly updated and adapted to the constantly changing clinical environment, which now includes the internet [4]. Developing guidelines alone would not be sufficient, as evidenced by the fact that 51% of US medical schools that reported incidents already had policies in place addressing online content [10]. Based on our findings, medical schools must integrate e-professionalism training into their curriculum. This refers to attitudes and behaviors that reflect traditional professionalism paradigms but are manifested through digital media [33]. These guidelines should not be restricted to patient privacy but must also emphasize respect and consideration for their professors, colleagues, and medical school. We recommend that medical schools (1) develop comprehensive e-professionalism guidelines, (2) implement mandatory training sessions on SM use, (3) regularly update curricula to reflect the evolving digital landscape and its impact on professional practice, (4) introduce regular audits and feedback sessions where students’ SM activities are reviewed and constructive feedback is provided, and (5) develop an anonymous reporting system for unprofessional behavior, ensuring students can report concerns without fear of retribution.

The limitations of our study include the use of a voluntary questionnaire that depended on self-reporting. Additionally, the generalizability of the findings may be limited due to the single institution sample and cultural context. The potential impact of self-reporting bias must be acknowledged, as participants might underreport unprofessional behavior. Moreover, this study did not account for other possible confounding variables such as the influence of peers or external SM trends. Two of the researchers are associate professors and 3 of them are students at the institution which may have influenced their study design and interpretation of results. Furthermore, we did not examine the specific content of medical students’ posts. We are, therefore, unaware if shared patient information followed Health Insurance Portability and Accountability Act guidelines and if posts positively or negatively depicted their school. Future studies should include content analysis of SM posts as that could provide deeper insights into the types of information shared and help identify specific areas for intervention. This analysis involves categorizing posts into themes such as educational content, patient confidentiality breaches, and professional interactions.

In conclusion, this study reveals significant gaps in medical students’ online behavior that can affect their medical schools’ image, patient care, and reputation. To foster students’ understanding of these issues, e-professionalism must be included in training curricula and assessments. This curriculum should include workshops, regular seminars on e-professionalism, and integration of SM conduct into existing ethics courses. Now, more than ever, medical schools should ensure that students develop a sense of belonging and pride in their institution and care about how it is represented worldwide. Information-sharing guidelines should strive to strike a balance between clinical knowledge sharing, protecting patients’ privacy, and reflecting an institution’s values and public image.

## Supplementary material

10.2196/57812 Multimedia Appendix 1 This is a copy of the survey.

## Abbreviation

KAU: King AbdulAziz University
MFMER: Mayo Foundation for Medical Education and Research
OR: odds ratio
SM: social media
