# Supplementary material for: Ethical Use of Social Media and Sharing of Patient Information by Medical Students at a University Hospital in Saudi Arabia: Cross-Sectional Survey
Source: JMIR Med Educ. 2025 Mar 24;11:e57812. doi: 10.2196/57812 (PMC11957465; doi:10.2196/57812)
Supplement: Multimedia Appendix 1 [file mededu-v11-e57812-s001.pdf]

* 1. Year of medical school:

- 1st year
- 2nd Year
- 3rd year
- 4th year
- 5th year
- 6th year
- Intern

* 2. Gender

- Male
- Female

* 3. Age:

- 18-20
- 21-25
- 26-30
- >30

* 4. Which social media platform do you use regularly (once a week)?

- Facebook
- Ticktock
- SnapChat
- Twitter
- Instagram
- Reddit
- Discord
- Telegram
- You have your own YouTube Channel
- None

* 5. Are your social media accounts Public or Private?

- Public- Anyone can see my whole profile
- Private-I choose who can see my profile
- Some are public and some are private
- I don't use social media

* 6. In your social media account, which of the following statements applies?

- [ ] I use my real name
- [ ] Profile image is a clear photo of myself
- [ ] I identify that I study at King Abdulaziz University
- [ ] I state that I'm a medical student
- [ ] None of the above

* 7. What have you used your social media account for

- [ ] Networking with other medical students/professionals around the world
- [ ] Keeping in touch with family/friends
- [ ] Providing medical advice and advocacy
- [ ] Entertainment
- [ ] Medical Education

* 8. Approximately, How much time do you spend on your social media accounts?

- Less than 1 hour/day
- 1 hour a day
- 2 hours a day
- 3 hours a day
- More than 3 hours a day

* 9. Have you ever checked your social media account while rounding on patients?

- Yes
- No

* 10. Have you ever posted a picture of a patient on social media without their permission?

- Yes
- No

* 11. Have you ever posted an image of part of a patient (including excised tumors or organs) or a radiographic image of a patient without a patient's permission?

- Yes
- No

* 12. Have you ever posted an image of a work colleague or senior without their permission?

- Yes
- No

* 13. Have you ever uploaded a video/image of a lecture or workshop online without the lecturer's permission?

- Yes
- No

* 14. Have you ever discussed an incident that happened in your institution online?

- Yes
- No

* 15. Have you ever discussed a patient you saw at your institution online?

- Yes
- No

* 16. Does your institution have a professional code of conduct or protocol that addresses the use of social media?

- Yes
- No

* 17. Did you receive any training during medical school or residency of the rules and regulations on the professional use of social media?

- Yes
- No

* 18. Have you ever experienced cyberbullying?

- Yes
- No

* 19. Which of the following applications do you use frequently to look up medical information?

- [ ] YouTube
- [ ] Wikipedia
- [ ] Medline
- [ ] Amboss
- [ ] UptoDate
- [ ] Osmosis

Other (please specify)
